# Effects of Steel Slag Powder and Expansive Agent on the Properties of Ultra-High Performance Concrete (UHPC): Based on a Case Study

**DOI:** 10.3390/ma13030683

**Published:** 2020-02-03

**Authors:** Shunkai Li, Shukai Cheng, Liwu Mo, Min Deng

**Affiliations:** 1 College of Materials Science and Engineering, Nanjing Tech University, Nanjing 21009, China; andymoliwu@njtech.edu.cn (L.M.); dengmin@njtech.edu.cn (M.D.); 2 CCCC Wuhan Harbor Engineering Design & Research Institute Co., Ltd., Wuhan 430000, China; chengsk@whut.edu.cn; 3 State Key Laboratory of Materials-Oriented Chemical Engineering, Nanjing 210009, China

**Keywords:** ultra-high performance concrete (UHPC), steel slag powder, expansive agent, compact stacking theory, total shrinkage, hydration process

## Abstract

In view of the performance requirements of mass ultra-high performance concrete (UHPC) for the Pang Gong bridge steel cable tower in China, the UHPC incorporating of steel slag powder and hybrid expansive agents is optimized and prepared. The effects of steel slag powder and hybrid expansive agents on the hydration characteristics and persistent shrinkage of UHPC are investigated. The results indicate that 15 wt.% steel slag powder and 5 wt.% hybrid expansive agents can effectively reduce the drying shrinkage deformation of UHPC with a slight decrease of strength. Heat flow calorimetry results show that the incorporation of steel slag powder and expansive agents decreases the hydration heat at three days. Moreover, the obtained adiabatic temperature rise of UHPC is 59.5 °C and the total shrinkage value at 180 days is 286 με. The hydration heat release changes of large volume UHPC in the steel-concrete section of cable tower is agreed with the result of adiabatic temperature rise in the laboratory.

## 1. Introduction

Ultra-high performance concrete (UHPC), as a new cement-based material, is different from traditional high strength concrete (HSC) and steel fiber reinforced concrete (SFRC), which has excellent mechanical and durability properties [1,2,3,4]. Normally, the compressive strength of UHPC is higher than 150 MPa for structural concrete [5,6,7] and shows outstanding performance [8,9,10]. UHPC is produced via optimizing the granular mixtures at very low water-to-binder ratio (w/b < 0.2), using high amount of binding materials and a certain amount of steel fibers [11,12,13,14]. Moreover, UHPC could satisfy the requirements of structural engineering with its own high strength, toughness and durability. Now it has evolved as a commercial concrete.

Owing to its outstanding mechanical properties and durability, UHPC has been widely used in thin concrete structure in China such as steel bridge deck pavement, wet joint, small prefabricated components, etc. [15]. However, due to the extremely low water-to-binder ratio of around 0.2 and the typically high binder content of 800–1200 kg/m^3^ in UHPC, the shrinkage of UHPC (usually the total shrinkage > 500 με) is greater than that of the conventional high performance concrete (HPC) [16,17,18]. Generally, the autogenous shrinkage of UHPC accounts for a larger proportion of total shrinkage, while the dry shrinkage is smaller. Such a great shrinkage developed at the early ages (autogenous shrinkage) can induce potential cracking of UHPC, and thus further reduce its mechanical properties and durability [19,20]. Therefore, the shrinkage problem of UHPC is one of the important factors restricting its engineering application, and has become a hot and difficult point in current research. Particularly, when the UHPC is used in mass concrete structures, the large shrinkage deformation accompanied with huge cement hydration heat liberation is usually caused, which seriously restricts the stability, reliability and sustainability of concrete structure.

To reduce the magnitude of shrinkage and cracking potential, several mitigation strategies have been developed to reduce the shrinkage of UHPC. For instance, cement was replaced at different levels by industrial by-products such as ground-granulated blast-furnace slag (GGBS) [21], fly ash (FA) [6,22,23,24], and rice-husk ash (RHA) [25]. The use of these industrial by-products in UHPC could not only increase the long-term strength, decrease hydration heat and the shrinkage, but also reduce the cost and environmental burden [26,27]. Nevertheless, the by-products such as FA and RHA are lack in some regions. Therefore, there is an increasing need to seek an effective substitute for cement in the wide range applications of UHPC. Recently, limestone powder (LP), a kind of supplementary cementitious materials, has been more and more used in the cement-based materials production due to its stable supply, ease of quality control and reasonable price, which contributes to reduction of cement content. Previous literatures reported that the LP not only played at physical role of filling effects but also participated in hydration reaction [28,29,30]. In addition, it is also found that the use of steel slag powder as a mineral admixture in concrete could improve the workability, retard the hydration and decrease the autogenous shrinkage of concrete at early ages [31]. While the addition of steel slag powder tends to have a negative effect on the mechanical properties and durability. Hence, there is a clear motivation to effectively optimize the content of steel slag powder and expansive admixture to obtain a suitable strength but lower shrinkage and hydration heat in UHPC.

Many researchers have already done some interesting investigations to reduce the shrinkage of UHPC with the addition of high-performance admixtures. It has been reported that the incorporation of calcium-sulfoaluminates-CaO based expansive agent (CSA-CaO EA) could effectively reduce autogenous shrinkage of UHPC by over 20% and show a better contribution to shrinkage compensation [32]. A study indicated that the addition of 7.5% EA and 1% shrinkage-reducing agent reduced the shrinkage by approximately 80% after 1 day [33]. At present, the ettringite based EA has been utilized to reduce the autogenous shrinkage and drying shrinkage of UHPC, but the addition of EA may reduce the workability of fresh concrete, increase the air content and decrease the strengths [34]. Therefore, both the mechanical and durability properties of UHPC containing EA should be carefully investigated.

This study is carried out to investigate the influence of steel slag powder (SSP) and expansive agents (EA) on the performance of UPHC, and then evaluate the feasibility of using the two minerals in UHPC in the main tower of Pang Gong Bridge (Xiangyang, China). The UHPC is designed to be used as the connecting material between the steel and concrete structures with a volume of 300 m^3^. Cement is replaced with LP at a substitution level of 15 wt.%. SSP and EA are added as replacements of cement by different proportions, respectively. The workability, mechanical properties, drying shrinkage, hydration heat, and adiabatic temperature rise of UHPC is investigated. Additionally, the on-site temperature monitoring of UHPC with added SSP and EA is also assessed.

## 2. Materials and Methods

### 2.1. Materials

In this study, UHPC is designed to gain a compressive strength higher than 120 MPa and a high flowability by adopting steel slag power and limestone powder. The cementitious materials used in this study were Class 42.5 Ordinary Portland cement (OPC, Huaxin Cement Co., Ltd., Huangshi, China), silica fume (SF, Southeast Star Technology Development Co., Ltd., Chengdu, China), unidentified superfine limestone powder (LP) and steel slag power (SSP, Wuhan Iron Group, Wuhan, China). The physical properties and chemical compositions of the cementitious materials are shown in Table 1. Continuously graded quartz sand (QS, 0.6–1.25 mm) and quartz powder (QP, 0–0.6 mm) are used as fine aggregates. A highly effective polycarboxylate superplasticizer (SP) (Wuhan Harbor Engineering Design & Research Institute Co. Ltd., Wuhan, China) with a solid content of 32% and a water reduction rate of 30% is also used in this paper. In order to reduce the shrinkage of UHPC, a hybrid magnesia expansive agent (EA, Wuhan Sanyuan Special Building Materials Co. Ltd., Wuhan, China) is used in this study. The hybrid magnesia expansive agent is a mixture of calcium oxide (CaO) and magnesium oxide (MgO) and the weight ratios of CaO and MgO based EA are 56.5% and 22.6%, respectively. Moreover, straight steel fibres (SSF) with 13 mm length and 0.22 mm diameter are utilized in the UHPC.

### 2.2. Experimental

#### 2.2.1. Mix Design of Concrete Skeleton

The UHPC mixtures are designed by using the modified Andreasen and Andersen model (A&A) model in according to the most closely packed principle [5,35]. The distribution coefficient (q) may be used to determine the proportions of fine and coarse particles in the mixture, of which the value is selected as 0.23 based on the available literature [36,37]. The UHPC mixtures are listed in Table 2. It can be found that the amount of SSP in the designed UHPC are 10 wt.%, 15 wt.% and 20 wt.%, respectively. Two addition dosage of EA, namely 5 wt.% and 8 wt.%, are added in the mixture.

**Figure 1:**
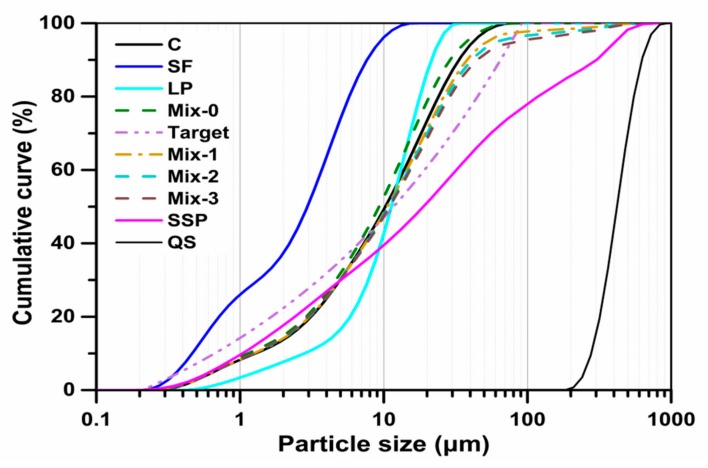
Particle size distributions (PSD) of the involved ingredients, the target and optimized grading curves of the UHPC mixtures.

The optimized grading curves of the designed mixtures are shown in Figure 1. Mix-1, Mixt-2 and Mix-3 denote the R1, R2 and R3, respectively. It can be observed that there is no difference between particle size distributions of the cementitious materials due to the fact that SSP and OPC have very similar particle size distributions.

#### 2.2.2. Flowability

The workability of the designed fresh UHPC with steel fibers is evaluated using flow test in accordance with the EN 12350-8, which are normally used for evaluating self-compacting concrete. Two diameters (perpendicular to each other) of the fresh UHPC are recorded and their average value is taken as the flowability.

#### 2.2.3. Mechanical Properties

The designed UHPC mixtures are cast in molds with the size of 100 × 100 × 100 mm, which are demolded 24 h after casting and then cured in water at the temperature of 20 ± 2 °C. The compressive strength of the samples is tested according to the Chinese standards GB/T 50081-2002. Three specimens are tested for each mixture.

#### 2.2.4. Total Shrinkage

According to the JTG E30-2005, the total shrinkage measurement of the designed UHPC mortar is conducted. Each group with three samples (size of 40 mm × 40 mm × 160 mm) is cured in standard environment with the temperature of 20 ± 2 °C and the relative humidity of 98 ± 2% for 3 days. And then they are placed in the drying room with a condition of temperature of 20 ± 2 °C and relative humidity of 60 ± 5%. The measurements of total shrinkage are conducted for 1 day, 7 days, 14 days, 28 days, 42 days, 56 days, 90 days, 120 days, 150 days and 180 days. The initial length of the specimens is L_0_ and the test length at the corresponding time is Lx.

#### 2.3.5. Hydration Heat

The prepared fresh paste is strictly designed according to the mix proportion and the rate of heat liberation and total heat within 3 days are recorded using an TAM AIR isothermal calorimetry. Then, the obtained results are normalized to the weight of the cement or binder.

#### 2.3.6. Adiabatic Temperature Rise

The fresh UHPC paste is added and vibrated, and then fulfilled in the 50 L cylindrical tin bucket. The adiabatic temperature rise of UHPC is carried out by using thermo-physical parameter measuring (NELD-TV810 Instrument, Beijing, China) in accordance with the Chinese standards CSL 352-2006. 

## 3. Results and Discussion

### 3.1. Slump Flow

**Figure 2:**
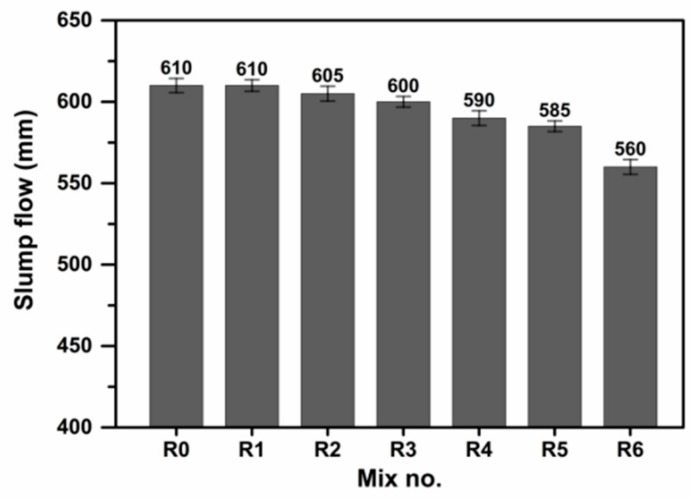
Slump flow of the designed UHPC.

The slump flow of the designed UHPC incorporated with SSP and EA is shown in Figure 2. It is observed that the slump flows of UHPC mixtures with 0%, 10%,15%, and 20% SSP are 610, 610, 605, and 600 mm, respectively. This is attributed to the fact that, when the SSP is used as supplementary cementitious materials (SCMs) in UHPC, it decreases the stress state between particles, aggregate and mortar, and therefore results in slight reduction of the slump flow [35]. In addition, the specific surface area of SSP is higher than that of cement, resulting in water demand increase, which also reduce the slump flow. Moreover, it is clear that the incorporation of EA exhibits negative effects on the slump flow of UHPC. In contrast to R0, the addition of 5 wt.% EA (R4) obviously declines the flow value of UHPC mixture. The reduction in flow slump may be related to the possible higher specific surface area of EA. While the combined addition of 15 wt.% SSP and 5 wt.% EA, similar trends are also observed in the test results. However, when the replacement level of EA is 8 wt.% (R6), the slump flow is reduced significantly, which reaches a minimum slump of 560 mm. Therefore, based on the obtained experimental results, it can be concluded that the incorporation of the EA at the dosages of 5 wt.% can achieve the optimal workability of UHPC.

### 3.2. Compressive Strength

**Figure 3:**
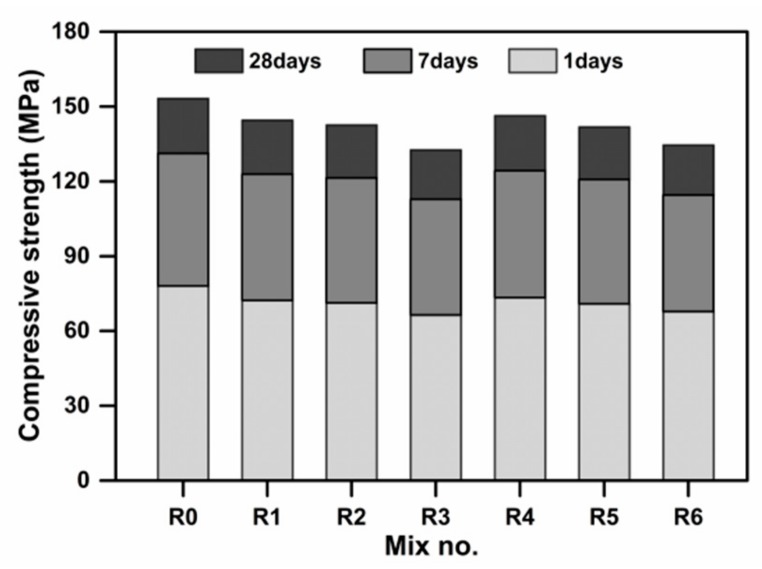
Compressive strengths of the designed UHPC at different curing ages.

The compressive strengths of the UHPC specimens at 1 day, 7 days, and 28 days are shown in Figure 3. It is evident that the compressive strength of UHPC gradually decreases with the increasing content of SSP. Compared to the sample R0, the strength decreasing ratios of samples at 1-day curing are 7.4%, 8.7%, and 15.0%, respectively. While the 28 days strengths of UHPC are decreased by 5.1%, 6.4% and 13.1%, respectively. When higher amount (20 wt.%) of SSP is utilized, the 28-day strength drops to 132.6 MPa. This is attributed to the lower reactivity of SSP with an activity index of 82.6% at 28 days, and thus makes very limited contribution to strength development of UHPC. During the early ages of hydration, the hydration activity of SSP is substantially lower than that of OPC. Moreover, the SSP has a retarding effect on the hydration of cement due to its lower reactivity [27]. Thus, the early compressive strength of UHPC is slight lower than the control group. However, with the hydration process continuing, the reactivity of SSP can provide suitable strength development of UHPC. Hence, to guarantee the compressive strength of the designed UHPC, it is advised to incorporate less than 15 wt.% SSP. Additionally, the results also indicate that the added EA has negative effects on the compressive strength. It is evident that, for the evaluated dosages, the inclusion of EA clearly decreases the strength. Similarly, the combination of SSP and EA induces the decrease in strength. It is noticed that the compressive strengths of R3 and R5 samples containing 15 wt.% SSP at 28 days are higher than 140 MPa, which remains acceptable.

### 3.3. Total Shrinkage

**Figure 4:**
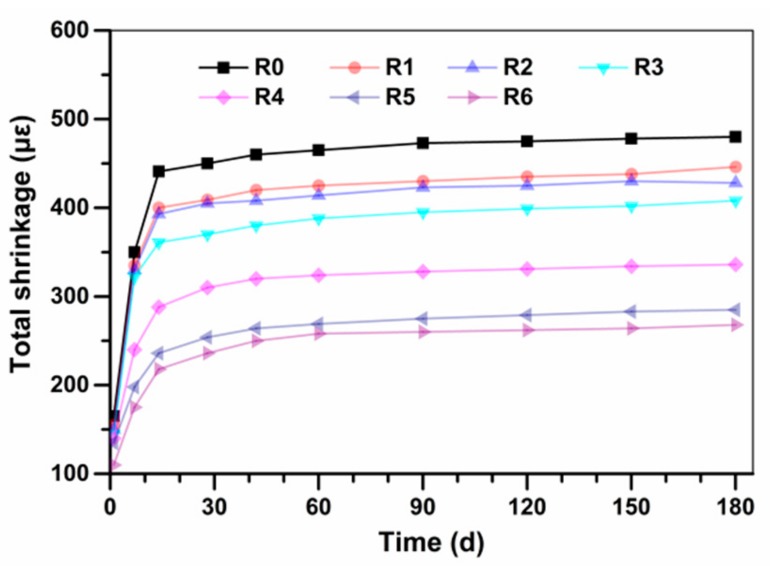
Total shrinkage development of the designed UHPC.

The total shrinkage of UHPC sample measured as a function of up to 180 days are presented in Figure 4. It can be seen that the shrinkage of UHPC in the first seven days is fast. The difference of total shrinkage of each sample is insignificant, and a clear distinction could be observed among all the UHPC mixtures at seven days. Afterwards, the increase rate gradually decreases, and the specimens continue to shrinkage within 14 days to 180 days. Due to the extremely low w/b in UHPC, the mixed water is quickly consumed at the early stage of hydration, inducing rapid decrease of relative humidity inside the system. In addition, outside moisture does not easily exchange with the inner moisture of UHPC due to the lower porosity and dense microstructure of UHPC [38,39]. As a result, the early shrinkage development of UHPC is comparatively rapid than the normal Portland cement concrete.

The shrinkage of all specimens is smaller than 500 με at the age of up to 180 days. The replacement of cement by SSP can not only reduce the cement content, but also restrain the shrinkage of UHPC. For instance, the drying shrinkage of UHPC added with 10%, 15% and 20% SSP at 180d are 446 με, 428 με and 408 με, respectively. Compared with UHPC without SSP, the total shrinkage of UHPC added with 10%, 15% and 20% SSP at 180 days are declined by 7.1%, 10.8% and 15.0%, respectively. The reason can be attributed to multiple reasons such as the shortening of early-age period of the concrete and the cement dilution effect [31]. In addition, both the accelerated cement hydration and the refinement of pore structure owing to the changed particle packing of binder can contribute to this result. Eventually, the total shrinkage of UHPC incorporating of SSP is lower than the control group.

The addition of EA is very effective in reducing total shrinkage of UHPC. The total shrinkage of UHPC (R4) added with 5% EA are approximately 40% lower than that without addition of EA. Replacement of cement by 15% SSP may further mitigate the total shrinkage of UHPC added with 5% EA. Moreover, the total shrinkage of UHPC decreases with the increasing content of EA. This might be attributed to the synergism effects of the combined use of CaO and MgO, which could contribute to the expansion and compensate the total shrinkage at both early and later ages, resulting a larger ultimate expansion [34]. Previous study suggested that the more stable hydration products from MgO is expected to perform a long-term expansive effect due to the formation of Mg(OH)_2_ nano-crystals.These nano-crystals recrystallized into large crystals to press the hardened binder paste and enlarge the microcracks existing in the paste, which thus causes expansion. However, when the EA amount is 8 wt.%, the compressive strength and flowability of UHPC are obviously decreased. Therefore, the suitable combination of SSP and EA can effectively reduce total shrinkage of UHPC without decreasing significantly the workability and mechanical properties.

### 3.4. Isothermal Calorimetry

**Figure 5:**
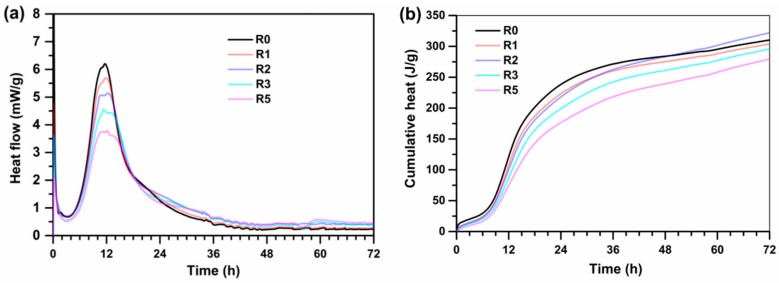
Hydration heat liberation of UHPC powder mixtures (paste): (**a**) normalized heat flow and (**b**) normalized total heat.

Figure 5 presents the hydration heat flow of several typical UHPC mixtures by using isothermal calorimetry. As shown in Figure 5a, the maximum heat flow peak of UHPC occurs at about 12 h and then decreases with the increasing content of SSP incorporated and with the addition of 5% EA. Consequently, the cumulative heat released from the UHPC pastes containing SSP is lower than that of the control group (Figure 5b). Due to the filler effect, the addition of SSP firstly reduces the cement content and suspends the hydration progress. However, in addition to the filler effects, there is a cement dilution effect, which increases the w/c ratio and provides more available water and space for the cement hydration. This decrease of cumulative heat is due to the decline in the absolute amount of cement involved in the hydration reaction [39]. Additionally, it is clear that the combined of SSP and EA exhibits the lowest hydration peak and total cumulative heat. Moreover, it is evident that that an additional peak is formed around the main hydration heat peak (Figure 5a). This phenomenon is attributed to the formation of mono/hemi-carboaluminate phases [40], which occurs and emits a large amount of heat in the system of UHPC containing LP.

### 3.5. Adiabatic Temperature Rise

**Figure 6:**
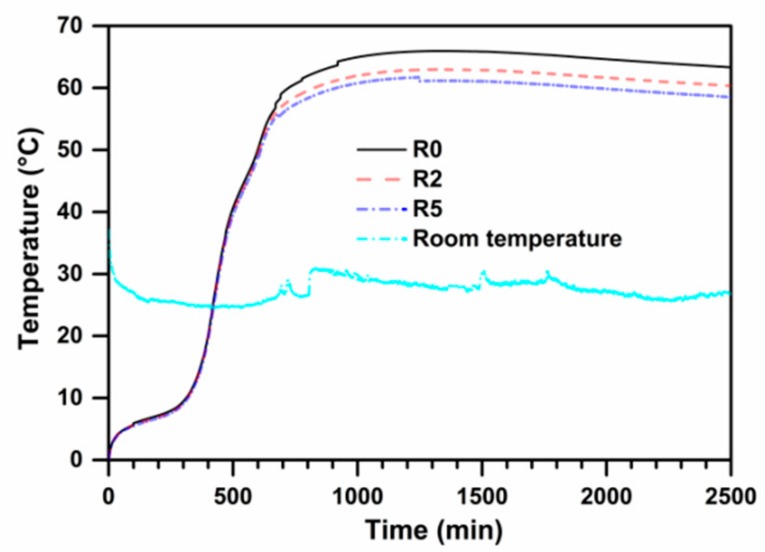
Adiabatic temperature rise curves of UHPC mixtures R0, R2 and R5.

The adiabatic temperature rise test of R0, R2 and R5 samples are presented in Figure 6. From the Figure 6, the adiabatic temperature rise of R0 sample initially increases and then reaches the maximum value. The curves of R2 and R5 samples show a similar tendency. It is also seen that the rate of temperature rise is fast firstly and then reaches a stable value, finally that of the temperature drops slowly until the tested time. During the testing time, the maximum temperature appears at time 1261 min (about 21 h), which means that the hydration heat of the concrete reaches its peak value at 21 h. The maximum adiabatic temperature rises are 65.5 °C, 62.9 °C, and 59.5 °C, respectively. These results indicate that the incorporation of SSP and EA in UHPC exhibit positive effects on the adiabatic temperature rise, namely reducing the released hydration heat liberation efficiently.

### 3.6. A Case Study of Mass UHPC for Bridge Steel Cable Tower

**Figure 7:**
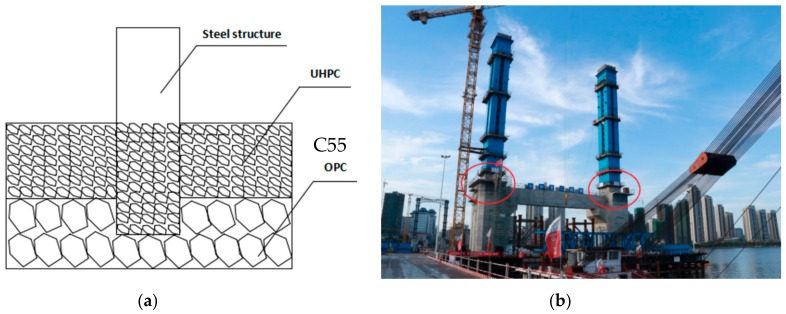
The schematic diagram (**a**) and construction on site (**b**) of mass UHPC for bridge steel cable tower.

**Figure 8:**
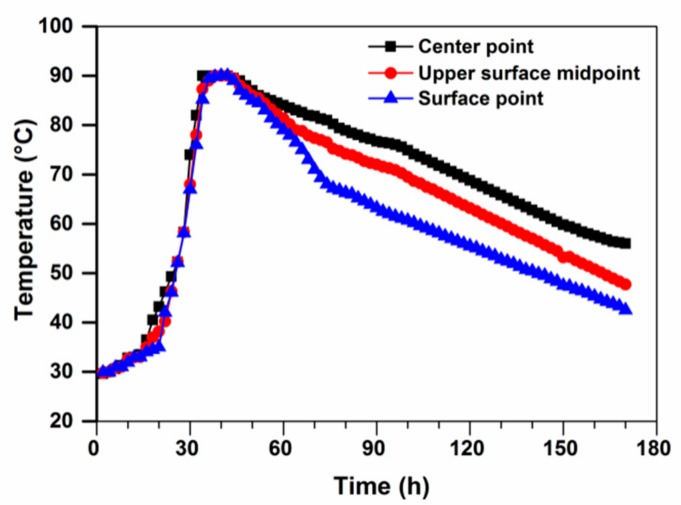
In-situ monitoring of the temperature rise in the mass UHPC.

The steel-concrete section of the cable tower of Peng Gong bridge was divided into two symmetrical zones. The dimension of each section was 6 × 10 × 2.5 m. The designed casting volume of UHPC was about 300 m^3^. The schematic diagram construction on site of mass UHPC section in bridge steel cable tower is shown in Figure 7. The workability and mechanical properties of UHPC by using field sampling are listed in Table 3. During the casting process, the temperature sensors were embedded in the center point, the upper surface midpoint (from surface to 0.625 m) and the surface point (from surface to 0.1 m), respectively. The measured temperature was demonstrated in Figure 8. The surface was continuously wet-cured and the side mold is kept insulation and humidification. It is clear that the monitored temperature peak in concrete appeared and reached 90 °C at 30 h after casting, the maximum adiabatic temperature rise was 61 °C, while the temperature difference between inside and outside was small during the whole monitoring process, and the maximum value was 15 °C at 72 h. At seven days, the temperature at center point and surface point both dropped to 56 °C, and the seven-day compressive strength of cored concrete reached 110 MPa. The on-site monitored temperature rise in the large volume UHPC of the tower steel-mixed section is consistent with the laboratory adiabatic temperature rise results. 

## 4. Conclusions

This study presents the effect of SSP and EA on the slump flow, compressive strength, drying shrinkage, and hydration heat of mass UHPC used in the steel-concrete section of a cable tower. Based on the obtained experimental results, the following conclusions can be drawn:

1. With the increase of SSP content, the workability and compressive strength of the designed UHPC decreases. This is mainly due to the addition of SSP decreases the stress state between particles, aggregate and mortar, which declines the slump flow and strength. The additional EA has a negative effect on the workability and mechanical properties of UHPC. The suitable contents of SSP and EA are 15 wt.% and 5 wt.%, respectively.
2. The incorporation of SSP and EA significantly reduces the total shrinkage and hydration release heat of UHPC. The total shrinkage at the age of 180 days is decreased by approximately 40%. Moreover, the adiabatic temperature rise of UHPC containing SSP and EA obtained from the experimental test is 59.5 °C, which is reduced by 6.1 °C compared with the control group.
3. The monitored temperature peak appears and reaches 90 °C at 30 h after casting, while the temperature difference between inside and outside is small during the whole monitoring process, the maximum adiabatic temperature rise is 61 °C and the maximum temperature difference is 15 °C, respectively. The heat release in the mass UHPC of the tower steel concrete section is consistent with the adiabatic temperature rise gained in laboratory.

## Figures and Tables

**Table 1:** Chemical compositions of the cementitious materials (%).

Chemical Composition	OPC	SF	LS	SSP
Al_2_O_3_	5.69	0.25	0.09	0.84
SiO_2_	21.27	94.65	0.41	12.67
CaO	60.15	0.36	54.75	48.66
MgO	2.41	0.47	2.61	2.80
Fe_2_O_3_	3.16	0.15	0.11	21.70
Na_2_O	0.14	0.13	-	-
K_2_O	0.69	0.84	-	-
SO_3_	3.66	0.69	-	-
L.O.I	3.95	2.29	39.9	8.59
Physical properties	-	-	-	-
Specific gravity (kg/m^3^)	3210	2300	2640	2810
Specific surface area (m^2^/kg)	380	3800	1200	600
Water demand ratio (%)	100	115	105	95

**Table 2:** Mix proportion of the designed UHPC.

No.	OPC (kg/m^3^)	SF (kg/m^3^)	LP (kg/m^3^)	SSP (kg/m^3^)	EA (kg/m^3^)	QP (kg/m^3^)	QS (kg/m^3^)	Water (kg/m^3^)	SP (kg/m^3^)	SSF (vol.%)
R0	700	100	200	0	0	150	850	160	18	2
R1	600	100	200	100	0	150	850	160	18	2
R2	550	100	200	150	0	150	850	160	18	2
R3	500	100	200	200	0	150	850	160	18	2
R4	665	100	200	0	35	150	850	160	18	2
R5	515	100	200	150	35	150	850	160	18	2
R6	494	100	200	150	56	150	850	160	18	2

**Table 3:** Performance of UHPC mass concrete used in the cable tower.

Slump Flow (mm)		Flexural Strength (MPa)			Compressive Strength (MPa)		
Zero Time	1 h	3 days	7 days	28 days	3 days	7 days	28 days
610	600	21.5	23.6	25.4	62.5	110.0	141.2
